# Development of PREPARE for Autistic Adults: An Adult Autism Training for Resident Physicians Designed with Autistic Adults and Family Members

**DOI:** 10.1089/aut.2023.0137

**Published:** 2025-02-05

**Authors:** Brittany N. Hand, Daniel Gilmore, Holden DeVassie, Anne Longo, Lisa Juckett, Christopher Hanks, Susan M. Havercamp, Daniel Coury

**Affiliations:** ^1^ School of Health and Rehabilitation Sciences, The Ohio State University College of Medicine, Columbus, Ohio, USA.; ^2^ Center for Autism Services and Transition, Columbus, Ohio, USA.; ^3^ Nationwide Children's Hospital, Columbus, Ohio, USA.

**Keywords:** health care, provider training, autism, autistic adults

## Abstract

One barrier to meeting autistic adults' health care needs is the dearth of physicians with autism training. We developed an adult autism training for residents, who are postdoctoral physicians training to become specialists, in internal medicine or family medicine. We used formative evaluation to design the training with autistic adults and family members of autistic adults, who were paid consultants. The training includes six prerecorded presentations, six case studies, and two standardized patient scenarios. We conducted focus groups and interviews with 23 residents and 14 faculty who educate residents. We described the curriculum, reviewed the content in one module, and obtained feedback on maximizing feasibility and scalability. Using semantic-level inductive rapid qualitative analysis we identified three themes and two subthemes. First, “flexibility is key” described ways to increase flexibility to accommodate resident and faculty schedules across programs. Second, “time is the most valuable asset” described the need to minimize duration and maximize impact. Third, “buy-in is necessary” described ways to increase buy-in from residents and residency leadership. Two subthemes, “we don't talk much about neurodivergence” and “this content applies to all patients,” describe how to increase buy-in by highlighting how this training fills a gap in resident education and can be generalized to multiple populations. Results highlighted ways to modify our training to maximize implementability across different residency programs. Next steps include pilot testing of feasibility, acceptability and effects on resident self-efficacy, attitudes/beliefs, and knowledge. In the long term, we expect this will yield more adult care physicians prepared to meet autistic adults' needs.

## Abstract (plain language summary)

Community brief

Why was this program developed?

Maximizing autistic adults' physical and mental health is important to autistic adults and their family members. However, too few physicians currently are willing and able to meet autistic adults' health care needs. Other researchers have found that disability trainings can improve physicians' self-efficacy in caring for people with disabilities, knowledge about disabilities and evidence-based practices, and willingness to provide care for people with disabilities. Our research team worked with autistic adults and family members of autistic adults to develop a training program for resident physicians, who are postdoctoral physicians training to become specialists in internal medicine or family medicine.

What does the program do?

The training aims to improve resident physicians' knowledge, self-efficacy, and attitudes and beliefs about caring for autistic adults. The training includes six prerecorded presentations, six case studies, and two mock clinical scenarios to be portrayed by autistic actors. The training is comprised of six modules: Contextual and conceptual frameworks, Patient-centered care, Clinical assessment, Team-based practice, Care over the lifespan, and Legal obligations.

How did the researchers evaluate the new program?

We conducted interviews and focus groups with 23 resident physicians and 14 faculty from 11 different resident physician training institutions. We identified changes that would maximize the chance that the training could be used in different residency programs in the future. The interviewer described the training and shared the clinical assessment module with participants. Then, the interviewer asked questions such as “How easy or difficult would it be to incorporate this into your residency program?” and “What would need to change to meet the needs of residents in your program?”

What were the early findings?

We identified three themes, one with two subthemes. First, “flexibility is key” described ways to accommodate resident and faculty schedules. Second, “time is the most valuable asset” described the need to minimize duration and maximize impact. Third, “buy-in is necessary” described ways to increase interest in delivering or participating in the training from residents and residency leadership. Two subthemes, “we don't talk much about neurodivergence” and “this content applies to all patients,” describe how to increase buy-in by highlighting how this training fills a gap in resident education and can be generalized across populations.

What were the weaknesses of this project?

Participants' feedback was based only on the overview provided by the interviewer and the clinical assessment module, which they reviewed. However, we felt previewing one module was sufficient to provide feedback about the feasibility of incorporating this training in their residency program.

What are the next steps?

Next, we will test the entire training with a small group of residents at one training institution. We will examine outcomes such as feasibility, acceptability, and appropriateness of the intervention. We will also examine whether residents complete all aspects of the training. This will help us further refine the training before doing a larger-scale trial.

How will this study help autistic adults now or in the future?

In the long term, we expect this study will increase the number of adult care physicians who are willing and able to provide care for autistic adults. Ultimately, we hope that this will improve autistic adults' access to high-quality care and, therefore, their health.

## Introduction

Maximizing autistic adults’ physical and mental health is a high-priority issue among autistic adults, their family members, and other key stakeholders.^1^ For example, relative to non-autistic peers, autistic adults are twice as likely to have cardiovascular disease or diabetes and three times as likely to have mood or anxiety disorders.^2^ These co-occurring health conditions are compounded by autistic adults' high rates of unmet health care needs^3^ due to barriers accessing quality primary care.^4,5^ One of the biggest barriers is the scarcity of adult care physicians with training in providing care for autistic adults.^6–9^

In the United States, aspiring physicians must first complete a bachelor's degree, which often includes about 4 years of coursework, before enrolling in a 4-year, doctorate-granting medical school.^10^ Medical school typically includes 2 years of coursework and 2 years of required clinical rotations. After medical school, aspiring physicians apply to receive postdoctoral clinical training in a certain specialty, called residency. Resident physicians have completed medical school and have earned a doctor of medicine or doctor of osteopathic medicine degree.

Funding for residency programs is derived from both public and private sources that support physician workforce development, with the federal government being the largest contributor.^11^ The length and structure of residency programs vary by specialty and the training institution, but training generally consists of providing clinical care to patients with some protected didactic time.

The Accreditation Council for Graduate Medical Education (ACGME), which oversees graduate medical education in the United States, does not specify any requirement common to all residency programs for disability training, except that resident physicians must demonstrate respect and responsiveness to diverse patient populations, including those with disabilities.^12^

However, in a recent letter,^13^ the United States National Council on Disability urged ACGME to require all accredited residency programs to “adopt and implement disability cultural competency training to ensure that physicians are prepared to meet the health needs of people with disabilities” and urged that such trainings incorporate the Core Competencies on Disability Health Education,^14^ which establish the baseline expertise for providing quality care for patients with disabilities. These core competencies were developed through a rigorous 2-year process to establish consensus among people with disabilities and other key stakeholders on essential learning objectives for disability health care education.^14^

In conceptualizing developing an autism training for adult care physicians, our research team chose to target the residency phase of medical education. The rationale behind this is that (1) we could strategically target residency programs that train specialists and primary care physicians who are likely to encounter autistic adults in their practice; (2) resident physicians have protected didactic time but fully independent practicing physicians (called “attending physicians” in the United States) do not. Strategically targeting residency programs has successfully been used to deliver autism training to residents in developmental and behavioral pediatrics.^15^

However, there is currently a lack of adult autism-specific training for resident physicians.^9^ This results in a workforce of physicians who have low self-efficacy in delivering care for autistic adults, have limited knowledge about autism in adulthood, and lack the flexibility needed for accommodating the communication and sensory needs of autistic adults, such as modifying standard care processes (e.g., longer appointment times).^4,5,9^ As a result, there is a nationally recognized need to better prepare the workforce of adult care physicians to be readied and capable of meeting the needs of autistic adults.^16–18^

To address this gap in resident physician education, our research team is partnering with an advisory board of autistic adults and family members of autistic adults to develop an adult-focused autism training for resident physicians called Promoting Residents' Excellence in Patient-centered cARE (PREPARE) for Autistic Adults. The objectives of this study are to (1) describe the initial development of the training content in collaboration with our advisory board and (2) identify ways to maximize the feasibility, acceptability, and scalability of the training, based on qualitative focus groups and interviews with resident physicians and faculty from multiple training institutions.

## Emerging Practice

### Description of PREPARE for Autistic Adults

**FIG. 1.**
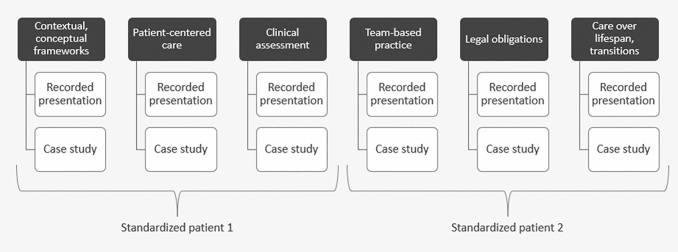
PREPARE for Autistic Adults training components.

This training is designed for residents in internal medicine (IM), family medicine (FM), and combined internal medicine and pediatrics (Med-Peds) programs. Our research team selected these residency programs because they train specialists and primary care physicians who will likely encounter autistic adults in their practice. We defined a priori that the training would consist of six modules, aligned with the six Core Competencies on Disability in Health Education (Fig. 1).^14^

In addition, our research team used the Blended Learning Model^19^ to inform this training by combining synchronous, asynchronous, and experiential learning. Trainings that are entirely synchronous^15,20,21^ may be more difficult to complete for resident physicians who often have irregular schedules.^22,23^ Yet, entirely asynchronous trainings do not allow for experiential learning, and thus are unlikely to result in changes in clinical practice.^24^

The training includes six short prerecorded presentations that can be viewed asynchronously, six case studies led synchronously by a facilitator, and two standardized patient scenarios designed to be portrayed by autistic adults who are paid for their time (Fig. 1). Supplementary Table S1 provides an overview of the content in the six modules. The training also includes a multiple-choice assessment that we developed in partnership with our advisory board to measure resident physicians' knowledge about delivering care for autistic adults.

### Community involvement in developing PREPARE for Autistic Adults

The initial development of this project was informed by the research priorities of the autistic community.^1^ In addition, the research team worked closely with our advisory board of autistic adults and family members of autistic adults, who are paid consultants on this project, to develop and refine the content included in the training. The PREPARE for Autistic Adults Advisory Board is a group author on this article and all board members are listed by name in the Acknowledgments section.

Board members were identified through wide distribution of an advertisement through multiple avenues at local, state, and national levels. The advisory board consists of six autistic adults and four family members (parents or grandparents) of autistic adults who were maximally diverse with a myriad of professional and lived experiences. One of the autistic adult advisory board members stopped working with us after a few months due to unforeseen schedule conflicts.

The research team worked closely with the advisory board to develop and refine the training content. The research team prepared prototypes of training components and distributed these to the board for review before each meeting. We reviewed two to four prototypes in each meeting and asked for suggestions to improve or change the content. This ensured that the training content included information that our advisory board felt was the most important for resident physicians to know to better meet the needs of autistic adult patients, challenge misconceptions about autistic people, and capture the diverse lived experiences of autistic adults.

The advisory board reviewed each training component twice. Changes based on advisory board feedback include, but are not limited to, examples provided in Supplementary Table S2.

## Evaluation Methods

### Study design

The research team conducted a semantic-level inductive rapid qualitative analysis to characterize factors that would impact the likelihood of widespread adoption of PREPARE for Autistic Adults in a variety of residency programs. We used this information to revise the training components based on resident and faculty input to maximize feasibility, acceptability, and scalability.

### Participants

We recruited residents and faculty from 14 residency programs across 11 training institutions. These residency programs are heterogeneous in (1) size (7–80 faculty; 16–120 residents); (2) medical school affiliation (public vs. private); (3) setting (academic vs. community medical centers); and (4) location (capital vs. major non-capital vs. small city).

We identified these recruitment sites by emailing IM, FM, and Med-Peds residency program directors or coordinators in Ohio and nearby states. Nine of the programs are in Ohio, two are in Pennsylvania, two are in Illinois, and one is in Georgia. Collectively, we recruited participants from six Med-Peds programs, five FM programs, and three IM programs. All residents and faculty members in these residency programs were eligible to participate.

Residency program directors or coordinators for the recruitment sites sent our study flier to residents and faculty through their internal listservs. Individuals who were interested in participating completed a brief REDCap form indicating their contact information and availability for a 1–2 hour virtual focus group or interview. Seventy-one residents and 19 faculty members expressed interest in the study. We recruited a maximum of two residents and two faculty from each residency program to ensure that feedback from no one residency program, particularly larger programs, would disproportionately influence our findings. As a result, 23 residents and 14 faculty participated in this study.

### Procedures

Participants completed a brief questionnaire to provide their demographic information, existing relationships with autistic people (including whether the participant identifies as autistic or neurodivergent), and our 27-item multiple choice knowledge assessment. Then, the first author conducted virtual focus groups consisting of two to six participants, with resident and faculty focus groups held separately, and one-on-one virtual interviews. We offered the one-on-one interview option because many potential participants had limited availability that did not readily align with other potential participants' availability. The first author (B.H.) conducted 7 focus groups and 12 interviews.

All data collection sessions started with B.H. providing an ∼20–25 minute overview of the PREPARE for Autistic Adults training, including previewing prototypes of the training components for the clinical assessment module. Next, B.H. asked participants open-ended questions about the training (e.g., “What are your thoughts about this training?”; “How easy or difficult would it be to incorporate this into your residency program?”; “What would need to change to meet the needs of residents in your program?”). The discussions lasted 20–51 minutes each (mean = 36 minutes), with focus group discussions lasting longer on average (mean = 46 minutes) than one-on-one interview discussions (mean = 30 minutes).

### Data analysis

We analyzed quantitative data with descriptive statistics to characterize the participants and evaluate responses to the knowledge assessment. We analyzed focus group and interview data with a semantic-level inductive rapid qualitative analysis using a postpositivist paradigm. Rapid qualitative analysis is a pragmatic approach that focuses on obtaining actionable targeted qualitative data to inform understanding of facilitators and barriers of an intervention.^25^

After prolonged engagement by conducting all data collection sessions, immersing herself in the transcripts, and holding regular debriefs with study team members, B.H. developed an initial codebook containing three themes: (1) flexibility is key; (2) “time is the most valuable asset”; and (3) buy-in is necessary. The third theme also had two subthemes with ways to increase buy-in: “we don't talk much about neurodivergence” and “this [content] applies to all patients.” Next, two coders (D.G. and H.D.) independently reviewed two transcripts and segments of text to these themes and considered the need to add new themes.

The coders met to discuss any new themes and update the codebook as needed. We calculated percentage agreement to guide our formative development of the codebook; for example, if we found low agreement (<90%) for a particular theme, we revised our codebook to clarify the definition of that theme. The team members independently used the updated codebook to revise all previously coded transcripts as needed, as well as code 4–5 new transcripts. This iterative process was repeated until the coders coded all transcripts.

In the final stages of the analysis, the research team reviewed the findings with the advisory board members to obtain their feedback on the changes proposed as a result of the focus group and interview findings. The research team also mapped our themes and subthemes to constructs from the Consolidated Framework for Implementation Research,^26^ a comprehensive taxonomy of factors that are likely to influence implementation of an intervention.

We conducted this final analysis stage for two reasons. First, this analytic approach allowed us to describe perceived barriers to and facilitators of implementing PREPARE for Autistic Adults, providing us with insight into factors that may impact future feasibility and outcomes. Second, we were able to identify additional changes to PREPARE for Autistic Adults we could make now to maximize future feasibility and likelihood of effectiveness.

### IRB approval

This study was reviewed and approved by the Institutional Review Board at The Ohio State University (#2022B0030).

## Results and Lessons Learned

Table 1 contains information about the research participants. When the research team examined responses to our prototype knowledge assessment, we identified 10 questions that at least 90% of residents answered correctly (Supplementary Data S1). As a result, we eliminated these items from the knowledge assessment, as they are unlikely to capture change in residents' knowledge over time.

**Table 1.** Participant Characteristics

	Residents (*n* = 23)	Faculty (*n* = 14)
Residency type, *n* (%)		
IM	6 (26.1)	2 (14.3)
FM	6 (26.1)	5 (35.7)
Med-Peds	11 (47.8)	7 (50.0)
Age category, *n* (%)		
21–30	16 (69.6)	0 (0.0)
31–40	7 (30.4)	7 (50.0)
41–50	0 (0.0)	5 (35.7)
51–60	0 (0.0)	2 (14.3)
Gender, *n* (%)		
Male	13 (56.5)	7 (50.0)
Female	9 (39.1)	6 (42.9)
Prefer not to answer, *n* (%)	1 (4.3)	1 (7.1)
Race, *n* (%)		
Asian^a^	8 (34.8)	4 (28.6)
Black	2 (8.7)	0 (0.0)
White	11 (47.8)	10 (71.4)
Prefer not to answer	2 (8.7)	0 (0.0)
Ethnicity, *n* (%)		
Hispanic or Latino	1 (4.3)	1 (7.1)
Not Hispanic or Latino	20 (87.0)	12 (85.7)
Prefer not to answer	2 (8.7)	1 (7.1)
Relationship to autistic people, *n* (%)		
Participant is autistic	0 (0.0)	0 (0.0)
Participant is neurodivergent	1 (4.3)	0 (0.0)
Immediate family member is autistic	2 (8.7)	3 (21.4)
Close friend is autistic	1 (4.3)	1 (7.1)
Extended family member is autistic	5 (21.7)	3 (21.4)
Casual friend is autistic	11 (47.8)	4 (28.6)
None of the above	8 (34.8)	6 (42.9)
Knowledge assessment score, median (IQR)	77.8 (70.4, 85.2)	85.2 (85.2, 88.9)
Revised knowledge assessment score,^b^ median (IQR)	61.1 (50.0, 66.7)	66.7 (66.7, 72.2)

^a^ Includes participants who reported race as Asian, Indian Asian, and South Asian.

^b^ After removing 10 items where at least 90% of residents answered correctly.

FM, family medicine; IM, internal medicine; IQR, interquartile range; Med-Peds, combined internal medicine and pediatrics.

All modules except contextual and conceptual frameworks had at least one item eliminated from the knowledge assessment (Supplementary Table S3). This resulted in a revised version of the knowledge assessment with 17 multiple-choice items. Our thematic analysis revealed three overarching themes and two subthemes, which are summarized in Table 2, and described as follows.

**Table 2.** Qualitative Analysis Findings on Ways to Maximize the Feasibility of PREPARE for Autistic Adults

Theme or subtheme	Description	Barriers and facilitators from CFIR	Changes to the training
Flexibility is key	The training needs to be flexible to be feasible with variable resident and faculty schedules and for residency programs with different structures.	Adaptability (facilitator)	• Can watch videos asynchronously or synchronously • Standardized patient can be telehealth or in person • Sample schedules for how the training could be delivered over different spans of time (e.g., 2 weeks vs. 8 weeks)
“Time is the most valuable asset”	Due to limited time that residents and faculty have, it is critical to streamline and shorten the training wherever possible while maximizing impact.	Available resources/time (barrier)	• Reduce video length from 20 to 15 minutes each • Allow video viewing at >1 × speed • Decrease total training time from 7 to 5 hours • Include “clinical pearls” at the end of all presentations
Buy-in is necessary	For successful adoption of the training, buy-in (from residents, from faculty, from leadership) is needed. Two subthemes hereunder highlight ways to improve buy-in.	Innovation recipients; innovation deliverers; relative priority; mission alignment; external pressure (facilitators)	• Offer a certificate of completion for resident resumes • Training should be opt-out vs. opt-in • Highlight relevance to board certifications
“We don't talk much about neurodivergence”	This training fills a gap in current residency education or is complimentary to existing, more informal education in this area.	Relative advantage (facilitator)	• Highlight training fills a gap in current education
“[This content] applies to all patients”	Although the training was designed to improve care for autistic adults, the lessons and skills in the training generalize to many patient populations.	Human equality and centeredness (facilitator)	• Highlight relevance to cultural competencies • Relevance to health disparities • Can apply to patients with other disabilities

CFIR, consolidated framework for implementation research.

### Flexibility is key

This theme described the need to maximize flexibility to facilitate accommodating for variable resident and faculty schedules across programs. One faculty member said, “I like the idea of having the flexibility so [the training is] more applicable to multiple programs.” Specific recommendations included having options for the prerecorded presentations to be viewed synchronously or asynchronously and standardized patient encounters to be conducted in person or through telehealth. Regarding flexibility for in-person or telehealth standardized patients, one resident said,

“You can have adaptations for both because a lot of programs may not have access to standardized patients in person or getting them on campus or to [a simulation] lab can also be challenging, or they may not have availability of persons on the spectrum who [are local and] would be willing to do it.”

Owing to heterogeneity in residency program schedules, one faculty member said, “I think [the duration of the training] might have to be tweaked based on the schedule of the particular residency.” Multiple participants recommended having sample schedules for programs to illustrate how PREPARE for Autistic Adults could be delivered in 2, 4, or 8 weeks.

### “Time is the most valuable asset”

This theme described the need to minimize duration and maximize impact of the training because limited faculty and resident time could be a barrier to implementing the training. Specific recommendations included reducing prerecorded presentation duration from 20 to 15 minutes, with one resident saying, “If it can be under 15 [minutes], that would probably get better turnout in terms of watching the whole thing.” Other participants commented on the total duration of the training, with faculty member saying, “I think seven hours is actually a big ask.”

In addition, multiple participants asked for “clinical pearls” or highly important takeaway points at the end of each presentation as well as for the prerecorded presentations to be hosted on a platform that would allow them to watch the video at >1 × speed.

### Buy-in is necessary

This theme described the need to maximize buy-in from residents, faculty, and residency leadership. This includes garnering support for implementing the training from faculty and leadership and ensuring that residents see the value in participating in the training. One recommendation for improving resident buy-in included offering residents a certificate of completion, which they can list on their resumes. One resident said:

“The only other thing that I would add is […] if they complete it, they can get a certificate or a credential that they can put on their resume that doesn't even have to be official or anything […] But it can easily be just, “blank has completed [autistic adult] sensitivity training” […] ‘cause that can also be something that people are always searching for an extra thing to put on their CV.”

Other participants recommended highlighting the relevance of this content to board certifications. Two subthemes, “we don't talk much about neurodivergence” and “[this content] applies to all patients,” describe how to increase buy-in by highlighting this training fills a gap in resident education and can be generalized to multiple populations.

#### We don't talk much about neurodivergence

This subtheme suggests buy-in may be facilitated by highlighting that this training fills a gap in current residency educational curricula. One faculty member said, “I was listening to the narrated PowerPoint and wishing that I had something like that when I was a resident.” Another faculty member said, “This kind of a training is exciting to me because I feel like it's a gap in the training I had, and I would love just practical tips while working with this population to better serve them.” Another faculty member said:

“I think that honestly this training is not just good for residents, but for practicing [physicians] as well because autism, the rates of patients diagnosed, has really increased and we're all taking care of autistic patients probably, or should be. And it would be good for everyone to be comfortable taking care of [autistic adult] patients.”

#### [This content] applies to all patients

This subtheme indicates another way to facilitate buy-in is to highlight the broader relevance of this training. One faculty member discussed how the training content, although developed to meet the needs specifically of autistic adults, may also be applicable to patients with disabilities more broadly saying, “I think there's similar challenges with [populations with other disabilities], especially types of […] disability that you can't necessarily see right when you see the person, but some of these concepts could apply to those populations as well.” Others commented the training highlights best practices in clinical care for all patients, including those with and without disabilities; one resident said:

“I think a lot of the recommendations in the [clinical assessment] module really aren't specific to autistic patients, it's really just best practice. Like, tell someone before you touch them and use language that is appropriate for them… So it's a good reminder of what best practice is, especially for [physicians] who don't have that as part of their practice.”

Another resident highlighted the relevance to cultural competencies, saying:

“I think [this content is] also applicable in lots of other patient scenarios. Starting with [autistic adults], I could see it as a good jumping off point. I think generally we learn on the fly how to [care for] with patients with […] different approaches to interactions with the physician. That includes language and religion and physical disabilities, et cetera and [autistic adults are] just one of many [such populations].”

## Discussion

Recent estimates suggest only 56% of physicians are willing to welcome patients with disabilities in their practice.^27^ In part, this is due to many physicians' poor self-efficacy^27^ in delivering care for this population. Indeed, less than half of physicians have high self-efficacy in their ability to provide quality care to people with disabilities.^27^ Disability trainings can improve health care providers' self-efficacy in caring for people with disabilities, knowledge about disabilities and evidence-based practices, and attitudes and beliefs about providing care for people with disabilities.^15,28–30^

The purpose of this study is to describe the initial development of an adult autism training for resident physicians and to obtain feedback on the training from faculty and residents. We are hopeful that this line of work and this training, after further testing and development, will ultimately increase primary care physicians' willingness to provide care for autistic adults and confidence in their ability to do so.

Results of our qualitative analysis revealed valuable suggestions for modifying the training to maximize the feasibility of implementation in the future. Based on feedback we received from faculty and residents, we decreased the duration of the prerecorded presentations and case studies (leading in a reduction of the total training duration from 7 to 5 hours), added “clinical pearls” to the end of each presentation, developed sample schedules for how the training could be implemented in different numbers of weeks, ensured the videos can be viewed at >1 × speed, and will offer a certificate of completion. These changes will be incorporated into the training for the next phase of this work, which will be a pilot test of PREPARE for Autistic Adults.

We recognize that feasibility and effectiveness testing are critical before more widespread implementation of this training. Our goal in the phase of the project described in this article was to gather formative stakeholder feedback so that, after pilot testing and further refinement, we would maximize the likelihood of successful implementation in the future. Indeed, initial small formative testing before more widespread implementation and systematic scaling-up is a key tenet of implementation science.^31^

We are currently carrying out pilot testing at The Ohio State University. We also recognize that a training that aims to improve physicians' knowledge, self-efficacy, and attitudes and beliefs about providing care for autistic adults will not solve all barriers to care experienced by autistic adults. Training nurses, medical assistants, and office staff who are also an integral part of the health care experience for autistic adults would require additional studies to develop or adapt trainings.

This study also does not offer solutions to structural barriers (e.g., time demands placed on practicing physicians) that may continue to limit the availability of primary care for this population. Additional studies and different intervention strategies would be necessary to address structural barriers to care. We have, however, embedded advice from our expert team on time demands and how residents may be able to manage them into this training.

## Conclusion

There is a critical need to increase the number of adult care physicians who are willing and able to meet autistic adults' health care needs. In this study, we described the initial development of a training aimed at better preparing IM, FM, and Med-Peds resident physicians to provide care for autistic adults. The training content was developed in partnership with autistic adults and family members of autistic adults.

The research team sought feedback on the training from faculty and residents from multiple training institutions and made changes to the training based on this feedback. Ultimately, we expect that these changes will increase the feasibility of residency programs using this training in the future, after effectiveness testing is completed and the training is further refined.
