# Supplementary material for: Development of PREPARE for Autistic Adults: An Adult Autism Training for Resident Physicians Designed with Autistic Adults and Family Members
Source: Autism Adulthood. 2025 Feb 5;7(1):112–20. doi: 10.1089/aut.2023.0137 (PMC11937756; doi:10.1089/aut.2023.0137)
Supplement: Supplementary Data S1 [file aut.2023.0137_suppl_materials1.docx]

**Supplemental material 1**: Items excluded from knowledge assessment due to >90% of resident physicians answering correctly.

1. Evidence shows that autistic adults who receive patient-centered care have
   1. Greater satisfaction with care
   2. Fewer inpatient hospitalizations
   3. Fewer emergency department visits
   4. All of the above [ correct answer ]
2. According to the Americans with Disabilities Act, all healthcare facilities are required to
   1. Ensure physical accessibility in terms of accessible parking spaces and entry, accessible exam rooms and bathrooms, and equipment
   2. Adjust policies, practices, and procedures to provide services, facilities, or accommodations
   3. Make communication, in all forms, easily understood
   4. All of the above [ correct answer ]
3. According to the Americans with Disabilities Act, what does “reasonable accommodation” mean for healthcare practices?
   1. The costs of accommodating a patient (e.g., longer appointment time) are passed on to the patients who use them
   2. Unless they would cause undue financial burden or would fundamentally alter the nature of the service, healthcare facilities are required to make changes to provide equal access to people with disabilities [ correct answer ]
   3. Instead of making expensive and disruptive modifications to their facilities, healthcare providers are allowed to refer patients with disabilities to accessible practices
   4. Only doctors who receive public funding and specialize in patients with disabilities are required to modify their practices to be accessible
4. You need to get a patient’s consent for a procedure but you have doubts about their ability to understand the procedure and make the decision independently. They are accompanied to the appointment by their sibling, who explains that the patient uses supported decision-making. What does this mean?
   1. The patient can make decisions on all matters independently or, if desired, in consultation with someone they trust [ correct answer ]
   2. You should talk to the sibling about the procedure; they can consent on the patient’s behalf
   3. For the patient’s safety, you should not offer the procedure without a guardian’s consent. Suggest that the sibling contact an attorney to establish guardianship
   4. To ensure that the patient has control, input, and an ability to make decisions affecting their lives to the greatest extent possible, you should ask the sibling to step out of the room while you talk to the patient about the procedure
5. Many autistic adults have complex physical and mental healthcare needs. Which of the following describes the best approach to meeting these needs?
   1. A single provider should address all of the patient’s mental and physical healthcare needs for consistency and predictability
   2. The primary care provider should refer their autistic adult patients to healthcare providers in other disciplines to ensure that the patient’s needs are met
   3. The primary care provider should address as many of the patient’s needs as possible while acknowledging when input from other disciplines would be best for the patient [ correct answer ]
6. An autistic adult who is non-speaking presents to clinic with their legal guardian for an annual physical exam. Which of the following would center the patient as the central member of their healthcare team?
   1. Explain to the patient what you’re going to do (e.g., looking in ears, listening to lungs) and check for consent before doing it
   2. Communicate primarily with the legal guardian since they ultimately will make healthcare decisions for the patient
   3. Use the method of communication (e.g., writing, verbal, pictures) that the patient understands best
   4. A and C [ correct answer ]
   5. A and B
7. A cis-gender autistic woman presents to your office for a routine physical. She has an intellectual disability and is accompanied by her father (legal guardian), but the patient communicates well throughout the visit. In the discussion, the patient asks to speak to you alone and reports she has recently become sexually active with a male partner. Which of the following are true?
   1. Her father should have been present for the entire discussion because he is the patients legal guardian, so you cannot keep information from him
   2. You should discuss contraception, testing for pregnancy and STI’s, and how to communicate the results to the patient [ correct answer ]
   3. She does not need cervical cancer screening because of her intellectual disability
   4. The patient should be offered permanent sterilization to ensure she does not get pregnant as she is unable to care for a baby
8. An autistic patient presents to you accompanied by their supporter, who is also their legal guardian. The patient’s supporter reports the patient is non-verbal and provides the history and answers all questions you ask while the patient is watching a video on their tablet. Which of the following is correct?
   1. Because the patient has a legal guardian, there is no need to seek involvement of the patient in the visit
   2. Because the patient is non-verbal, they do not have capacity to understand their medical needs.
   3. Address the patient as they may understand everything you are saying, even if they don’t respond [ correct answer ]
   4. You should ask the supporter and to stop speaking while you engage with the patient
9. Which of the following would NOT be a reasonable accommodation for an autistic patient?
   1. Telling a patient you will only see them via telehealth because they became agitated at a past visit and made staff uncomfortable [ correct answer ]
   2. Providing a quiet room for the patient to wait in if the regular waiting room result in overstimulation
   3. Scheduling a patient in the 1^st^ time-slot of your day to avoid the potential of a long wait time
   4. Allowing a support person to accompany them for a procedure in an area that family are typically not allowed in
10. During an evaluation of an autistic patient, you realize they are due for several vaccines and need labs to monitor effects of a medication they are on for high blood pressure. When you bring this up, the patient explains they are afraid of needles and becomes notably anxious and declines both the vaccines and the labs. What is the next best step?
    1. You should stop their medication because they will not allow monitoring to ensure safety
    2. You should explore what has worked for them in the past to complete vaccinations and venipuncture
    3. You should send them to the Emergency Department to be sedated in order to complete the vaccines and labs
    4. You should discuss a personalized strategy of doing one per visit or returning to complete them at a future time when they are mentally prepared for this
    5. A and B
    6. B and D [ correct answer ]
