# Supplementary material for: Development of PREPARE for Autistic Adults: An Adult Autism Training for Resident Physicians Designed with Autistic Adults and Family Members
Source: Autism Adulthood. 2025 Feb 5;7(1):112–20. doi: 10.1089/aut.2023.0137 (PMC11937756; doi:10.1089/aut.2023.0137)
Supplement: Supplementary Table S1 [file aut.2023.0137_suppl_tables1.docx]

**Supplemental Table 1:** PREPARE for Autistic Adults modules and example content

| **Module** | **Questions addressed in presentation** | **Example resources and references** | **Case study key learning points** |
| --- | --- | --- | --- |
| Contextual & conceptual frameworks | - What is the prevalence of autism and how heterogeneous are autistic adults? - How do the medical and social models of disability differ? - What are neurodiversity affirming approaches to care? | - Article on defining & avoiding ableism & ableist language; contrasting social & medical models of autism^1^ - Autistic SPACE: A novel framework for meeting the needs of autistic people in healthcare settings^2^ - Book Unmasking Autism^3^ | - Understand that autism is a nonlinear spectrum with a great deal of heterogeneity - Apply neurodiversity affirming approaches to care to promote a supportive and safe healthcare environment for autistic adults |
| Professional, patient-centered care | - What are the dimensions of patient-centered care? - How can we apply patient-centered care with autistic adults? - How is patient-centered care beneficial for autistic adults? | - Autistic SPACE: A novel framework for meeting the needs of autistic people in healthcare settings^2^ - “Respect the way I need to communicate with you” article on autistic adults’ healthcare experiences^4^ - Articles on patient-centered care^5–7^ - Articles demonstrating impact of patient-centered care on autistic adults’ health^8–11^ | - Identify approaches to modifying the medical environment to better meet autistic adults’ needs. - Outline ways the physician and office staff can minimize barriers to health care visits difficult for autistic adults. - Recognize the importance of communicating directly with autistic adult patients in providing patient-centered care. |
| Clinical assessment | - What challenges faced by autistic adults may impact clinical assessment? - How can patient-centered care and accommodations be used during clinical assessment? - How can autism impact the presenting signs and symptoms of health conditions? | - Integrating autistic young adults in healthcare^20^ tip sheet - Book chapter on clinical assessment considerations^21^ - “Respect the way I need to communicate with you” article on autistic adults’ healthcare experiences^4^ - AASPIRE Toolkits^22^ for autistic adults & healthcare providers | - Identify approaches to overcoming barriers in completing an assessment of a patient who is unable to express their symptoms. - Describe possible causes of changes in behavior in a patient who is unable to provide a verbal history - Be familiar with health issues that are common among autistic people. |
| Legal obligations | - What barriers to healthcare impact autistic adults? - What are the legal obligations of healthcare providers to meet the needs of disabled patients? - What legal, formal, and informal options are available to support healthcare decision-making? | - Article on self-determination for autistic people^12^ - Healthcare & the Americans with Disabilities Act resource^13^ - Autistic SPACE: A novel framework for meeting the needs of autistic people in healthcare settings^2^ - “Respect the way I need to communicate with you” article on autistic adults’ healthcare experiences^4^ - Book section on supported-decision making for autistic adults^14^ | - Understand how accommodations improve the healthcare experience for autistic patients - Identify accommodations and their relationship to characteristics of autism - Recognize the role and range of supported decision making in health care |
| Team-based practice | - What services and providers could play a role in autistic adults’ health? - How can we center the patient as the central member of the healthcare team? - How can we use evidence-based practice to support shared decision-making? | - Article on care coordination for autistic patients^15^ - Resources on interprofessional team collaboration^16,17^ - The SHARE approach to shared decision-making^18^ - Articles on interprofessional collaboration in caring for autistic people^16,19^ | - Identify and work with providers in other disciplines to meet autistic adults’ healthcare needs - Apply shared decision-making strategies with autistic adults - Recognize that the patient is the central member of the healthcare team |
| Care over lifespan & during transitions | - What disparities are faced by autistic adults and how can we address them? - What changes as autistic adults move into and through adulthood? - How can we improve access to age-appropriate healthcare and health education? | - AASPIRE Toolkits^22^ for autistic adults & healthcare providers - Articles on co-occurring conditions among autistic adults and older adults^23,24^ - Article on mortality in autistic people^25^ - Articles on sexuality, gender identity & relationship education in autism^26–28^ | - Understand how being autistic may impact healthcare needs through different phases of adult life. - Identify opportunities for healthcare providers to decrease healthcare disparities faced by autistic adults. - Recognize that autism does not negate the need for routine care, including preventative care. - Improve comfort in addressing healthcare needs for autistic adults, with a particular focus on mental health, sexual health, and identification and treatment of common medical problems that convey increased health risks as people age. |

**Supplemental material references**

1. Bottema-Beutel K, Kapp SK, Lester JN, Sasson NJ, Hand BN. Avoiding Ableist Language: Suggestions for Autism Researchers. *Autism Adulthood*. Published online September 2, 2020. doi:10.1089/aut.2020.0014

2. Doherty M, McCowan S, Shaw SC. Autistic SPACE: a novel framework for meeting the needs of autistic people in healthcare settings. *Br J Hosp Med*. 2023;84(4):1-9. doi:10.12968/hmed.2023.0006

3. Price D. *Unmasking Autism: Discovering the New Faces of Neurodiversity*. Harmony Books; 2022.

4. Nicolaidis C, Raymaker DM, Ashkenazy E, et al. “Respect the way I need to communicate with you”: Healthcare experiences of adults on the autism spectrum. *Autism*. 2015;19(7):824-831. doi:10.1177/1362361315576221

5. Hughes R. Overview and Summary: Patient-Centered Care: Challenges and Rewards. *OJIN Online J Issues Nurs*. 2011;16(2). doi:10.3912/OJIN.Vol16No02ManOS

6. McCance T, McCormack B, Dewing J. An exploration of person-centredness in practice. *Online J Issues Nurs*. 2011;16(2):1.

7. NEJM Catalyst. What Is Patient-Centered Care? *Catal Carryover*. 2017;3(1). doi:10.1056/CAT.17.0559

8. Hand BN, Coury DL, Darragh AR, et al. Patient and caregiver experiences at a specialized primary care center for autistic adults. *J Comp Eff Res*. 2020;9(16):1131-1140. doi:10.2217/cer-2020-0155

9. Hand BN, Coury DL, White S, et al. Specialized primary care medical home: A positive impact on continuity of care among autistic adults. *Autism*. 2021;25(1):258-265. doi:10.1177/1362361320953967

10. Hand BN, Gilmore D, Coury DL, et al. Effects of a Specialized Primary Care Facility on Preventive Service Use Among Autistic Adults: a Retrospective Claims Study. *J Gen Intern Med*. Published online January 19, 2021. doi:10.1007/s11606-020-06513-7

11. Hand BN, Gilmore D, Harris L, et al. “They Looked at Me as a Person, Not Just a Diagnosis”: A Qualitative Study of Patient and Parent Satisfaction with a Specialized Primary Care Clinic for Autistic Adults. *Autism Adulthood*. Published online June 14, 2021. doi:10.1089/aut.2020.0082

12. Ward MJ, Meyer RN. Self-Determination for People with Developmental Disabilities and Autism: Two Self-Advocates’ Perspectives. *Focus Autism Dev Disabil*. 1999;14(3):133-139. doi:10.1177/108835769901400302

13. ADA National Network. Health care and the Americans with Disabilities Act. ADA National Network: Information, Guidance, and Training on the Americans with Disabilities Act. Published 2020. Accessed July 30, 2021. https://adainfo.us/healthcare

14. Bedard R, Hecker L, eds. Options for Adulthood: How to Support Decision-Making and Independence for People with Autism. In: *A Spectrum of Solutions for Clients with Autism Treatment for Adolescents and Adults.* Routledge; 2020.

15. Parker ML, Diamond RM, Guercio ADD. Care Coordination of Autism Spectrum Disorder: A Solution-Focused Approach. *Issues Ment Health Nurs*. 2020;41(2):138-145. doi:10.1080/01612840.2019.1624899

16. Bobbette N, Ouellette-Kuntz H, Tranmer J, Lysaght R, Ufholz LA, Donnelly C. Adults with intellectual and developmental disabilities and interprofessional, team-based primary health care: a scoping review. *JBI Evid Synth*. 2020;18(7):1470-1514. doi:10.11124/JBISRIR-D-19-00200

17. Bowman KS, Suarez VD, Weiss MJ. Standards for Interprofessional Collaboration in the Treatment of Individuals With Autism. *Behav Anal Pract*. Published online May 3, 2021. doi:10.1007/s40617-021-00560-0

18. Agency for Healthcare Research and Quality. The SHARE Approach—Essential Steps of Shared Decisionmaking: Quick Reference Guide. Published March 2023. Accessed October 3, 2022. https://www.ahrq.gov/health-literacy/professional-training/shared-decision/tools/resource-1.html

19. Bowman KS, Suarez VD, Weiss MJ. Standards for Interprofessional Collaboration in the Treatment of Individuals With Autism. *Behav Anal Pract*. 2021;14(4):1191-1208. doi:10.1007/s40617-021-00560-0

20. Hanks C, Cooley WC. Integrating Young Adults with Autism Spectrum Disorder into Your Practice: Tips for Adult Health Care Clinicians. Published online August 2019. Accessed April 13, 2020. https://www.gottransition.org/resourceGet.cfm?id=516

21. Crapnell T, Lau L, Hanks CD, Nicolaidis C, Kuo A. Autism. In: Pilapil M, DeLaet D, Kuo A, Peacock C, Sharma N, eds. *Care of Adults with Chronic Childhood Conditions: A Practical Guide*. Springer International Publishing; 2016:53-66.

22. Nicolaidis C, Raymaker D, McDonald K, et al. The Development and Evaluation of an Online Healthcare Toolkit for Autistic Adults and their Primary Care Providers. *J Gen Intern Med*. 2016;31(10):1180-1189. doi:10.1007/s11606-016-3763-6

23. Croen LA, Zerbo O, Qian Y, et al. The health status of adults on the autism spectrum. *Autism*. 2015;19(7):814-823. doi:https://doi.org/10.1177/1362361315577517

24. Hand BN, Angell AM, Harris L, Carpenter LA. Prevalence of physical and mental health conditions in Medicare-enrolled, autistic older adults. *Autism*. 2020;24(3):755-764. doi:10.1177/1362361319890793

25. Hirvikoski T, Mittendorfer-Rutz E, Boman M, Larsson H, Lichtenstein P, Bölte S. Premature mortality in autism spectrum disorder. *Br J Psychiatry*. 2016;208(3):232-238. doi:10.1192/bjp.bp.114.160192

26. Sala G, Hooley M, Attwood T, Mesibov GB, Stokes MA. Autism and Intellectual Disability: A Systematic Review of Sexuality and Relationship Education. *Sex Disabil*. 2019;37(3):353-382. doi:10.1007/s11195-019-09577-4

27. George R, Stokes MA. Gender identity and sexual orientation in autism spectrum disorder. *Autism Int J Res Pract*. 2018;22(8):970-982. doi:10.1177/1362361317714587

28. Pecora LA, Hooley M, Sperry L, Mesibov GB, Stokes MA. Sexuality and Gender Issues in Individuals with Autism Spectrum Disorder. *Child Adolesc Psychiatr Clin N Am*. 2020;29(3):543-556. doi:10.1016/j.chc.2020.02.007
