# Supplementary material for: Development of PREPARE for Autistic Adults: An Adult Autism Training for Resident Physicians Designed with Autistic Adults and Family Members
Source: Autism Adulthood. 2025 Feb 5;7(1):112–20. doi: 10.1089/aut.2023.0137 (PMC11937756; doi:10.1089/aut.2023.0137)
Supplement: Supplementary Table S2 [file aut.2023.0137_suppl_tables2.docx]

**Supplemental Table 2.** Example changes to training recommended by advisory board

| **Training Component** | **Example Changes** |
| --- | --- |
| Presentations | - Changing images in the presentation to capture concepts and lived experiences they were intended to portray more accurately - Changing the order in which information is covered - Add or expand content of high importance (e.g., diagnostic overshadowing, transitioning to older adulthood, sexuality and reproductive wellness) - Language considerations (e.g., change “preferred communication style” to “communication needs”) - Expand list of community resources (include Centers for Independent Living, Regional Centers) |
| Case studies | - Add a picture and demographic information to contextualize and humanize the autistic adult in each case - Add scenario where autistic adult wants to discontinue psychiatric medications - Discuss how providers can advocate for patients’ rights under ADA (e.g., extended appointment time) - Add responsibilities and expectations of the provider in a contingency plan for emergency medical situations |
| Standardized patient scenarios | - Clarify instructions for standardized patient - Add examples of what the actor can say if certain situations arise - Explicitly state type of decision-making support provided by supporter |
| Knowledge assessment | - Use gender neutral pronouns for all items unless sex assigned at birth or gender is relevant to the question |
