# Supplementary material for: Development of PREPARE for Autistic Adults: An Adult Autism Training for Resident Physicians Designed with Autistic Adults and Family Members
Source: Autism Adulthood. 2025 Feb 5;7(1):112–20. doi: 10.1089/aut.2023.0137 (PMC11937756; doi:10.1089/aut.2023.0137)
Supplement: Supplementary Table S3 [file aut.2023.0137_suppl_tables3.docx]

**Supplemental Table 3.** Number of items in knowledge assessment by module before and after removing items that over 90% of resident physicians answered correctly

| **Module** | **Original # of items** | | **Revised # of items** | |
| --- | --- | --- | --- | --- |
| Contextual & conceptual frameworks | 5 | 5 | |  |
| Professional, patient-centered care | 5 | 4 | |  |
| Clinical assessment | 5 | 2 | |  |
| Legal obligations | 5 | 2 | |  |
| Team-based practice | 4 | 3 | |  |
| Care over lifespan & during transitions | 3 | 1 | |  |
| TOTAL | 27 | 17 | |  |
